# The immunogenicity and safety of adenoviral-based vaccines

**DOI:** 10.1097/ACI.0000000000001167

**Published:** 2026-05-14

**Authors:** Joshua Gardner

**Affiliations:** Centre for Drug Safety Science, Department of Pharmacology and Therapeutics, University of Liverpool, Liverpool, UK

**Keywords:** adenovirus, COVID-19, T-cells, vaccines

## Abstract

**Purpose of review:**

Human adenoviruses (HAdVs) have shown promise as versatile and effective delivery vectors for vaccine development. This was highlighted during the COVID-19 pandemic, which demonstrated the importance of effective vaccines but also provided scope to explore potential limitations of current viral-vector based strategies. This review summarizes the current applications of adenoviral vectors, in addition to discussing the immunological mechanisms underpinning immunogenicity and safety of viral vector-based vaccines.

**Recent findings:**

Clinical trials involving HAdVs have been undertaken to combat a range of infectious diseases, such as SARS-CoV-2, Ebola and HIV-1. However, empirical evidence indicates that preexisting T-cell immunity to adenoviruses may occur independent of serological exposure, predominantly arising from the conserved nature of immunogenic hexon proteins. As a result, T-cell responses are frequently detectable and often demonstrate broad cross-reactivity between human and nonhuman adenovirus (AdV) serotypes. Adverse events to vaccination are rare, although thrombotic events and severe cutaneous adverse reactions have been reported.

**Summary:**

Antivector T-cell immunogenicity arising from preexisting viral exposure or T-cell cross-reactivity may influence vaccine-specific immune responses. Continuous iterative refinement within the development of new vaccine vector technologies is therefore essential to circumvent limitations associated with vector-specific T-cell responses. However, mechanistic insight on the role of T-cells within pathomechanisms of adverse events remains unclear.

## INTRODUCTION

HAdVs are large (>90 nm), nonenveloped double stranded DNA viruses that are 34–36 kbp in length and arranged in an icosahedral capsid shell made up of at least 12 distinct virion proteins [1,2]. Although immunological epitopes can be diverse, HAdVs possess a universal basic capsid structure consisting of a hexon protein, fibre knob and penton base [1]. Over 100 HAdV genotypes have been identified through whole genome sequencing (WGS), belonging to 51 serologically distinct groups and classified into seven species (A–G) [3,4]. Human adenovirus serotype 5 (HAdV-5; Species C) is the most comprehensively studied HAdV and has formed the backbone of vaccine and oncolytic vector development. Despite extensive research into its use as a vector candidate due to its potent immunogenicity, several limitations of HAdV-5 have now been recognised with respect to vaccine development. High seroprevalence in some populations (60–98%) leave HAdV-5 susceptible to critical immunological drawbacks driven by preexisting immunity, including, but not limited to, the induction of antivector T-cell responses and the presence of neutralizing antibodies that can attenuate vector efficacy [5,6].

Immunological memory to common adenoviruses is widespread, which can weaken vaccine and transgene-specific responses or cause unwanted, off-target immune activation that have the potential to impede vaccine efficacy and cause immune-related adverse events [7,8]. In the context of COVID-19 vaccination, a range of HAdVs and non-human AdV vectors were deployed, including HAdV-5 (Ad5-nCoV; Convidecia), HAdV-26 (Ad26.COV2.S; Janssen/J&J) and chimpanzee adenovirus (ChAdOx1 nCoV-19; AZD1222) [9–11]. Such platforms were selected due to a low seroprevalence in humans, with the aim of circumventing preexisting immunity and providing robust anti-transgene immunogenicity. 

**Box 1:**
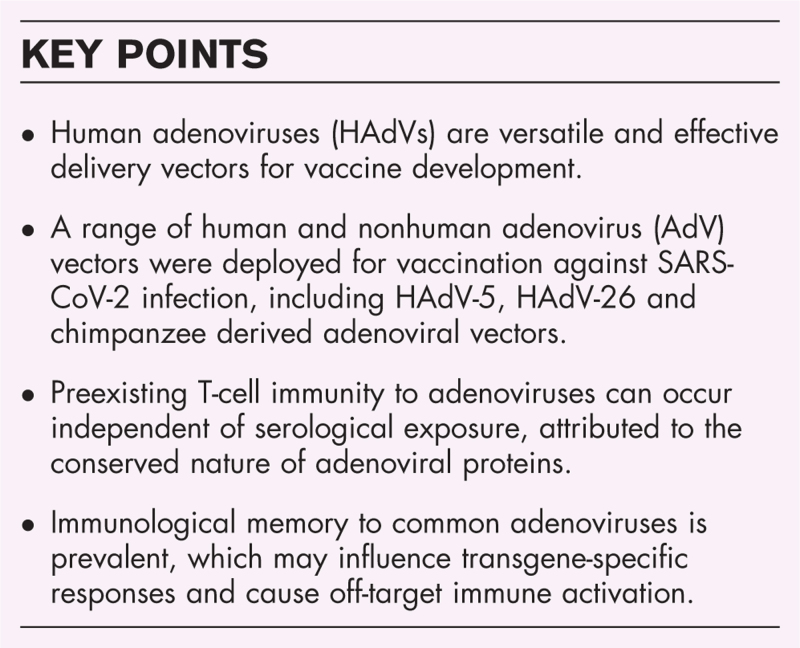
no caption available

### Current applications of viral-vector vaccines and therapies

The COVID-19 pandemic highlighted the importance of effective vaccines, with ChAdOx1 nCoV-19, Ad26.COV2.S and Ad5-nCoV the most widely deployed adenoviral-based platforms following the administration of >3 billion doses globally [12]. These platforms utilise first generation adenoviral vectors, defined by deletion of E1 and/or E3 genes to render the vector replication-incompetent [13]. Viral genes necessary for replication are replaced with a transgene encoding stabilized SARS-CoV-2 Spike (S) proteins which induce robust T-cell (CD4^+^/CD8^+^) and B-cell (antibody) responses following vector-mediated transduction into target cells [10,14,15]. In ChAdOx1 nCoV-19, the ChAdOx1 vector was derived from the Y25 chimpanzee adenovirus (ChAdY25) and primary efficacy data from randomized clinical trials demonstrated overall vaccine efficacy of 70–75% for the prevention of symptomatic disease [16,17]. In contrast, Ad26.COV2.S developed by Janssen Pharmaceuticals, incorporated a HAdV vector (HAdV-26; Species D) for transgene delivery and was administered as a single-dose vaccine that was shown be 66% effective for the prevention of mild-moderate COVID-19 cases and 85% effective against severe disease [18]. Ad5-nCoV, also known as Convidecia, was first approved for use in China and widely administered throughout Asian populations [9]. Significantly, Ad5-nCoV represents the only licensed, single-dose HAdV-5 vectored COVID-19 vaccine and demonstrated 57% efficacy against symptomatic COVID-19 [19]. It is possible that the lower efficacy of Ad5-nCoV may stem from preexisting T-cell immunity to HAdV-5 and dominant antiviral immunogenicity, with aerosolized booster doses later introduced to elicit mucosal immunity and negate the impact of undesirable vector-driven immunological memory [20].

Adenoviral-based vaccination platforms have been explored to combat a range of emerging infectious diseases outside of SARS-CoV-2. The STEP study, conducted by Merck, evaluated the use of a replication-incompetent HAdV-5 vectored HIV-1 vaccine (MRKAd5 HIV-1 gag/pol/nef) and represented a watershed moment in adenoviral vaccine development. Crucially, clinical trials were halted, not only due to a lack of efficacy, but following the highly unexpected finding of increased viral titres and HIV-1 infectivity rates in vaccinated volunteers compared with placebo groups [21]. Although immunological rational for this result has not been fully explained, HIV-1 infection was found to be higher in HAdV-5 seropositive individuals and suggestive of preexisting immunity from HAdV-5-specific CD4^+^ T-cells influencing the cellular dynamics of targeted HIV infection [22]. More recently, research focus has shifted toward the incorporation of rare serotypes, nonhuman adenoviruses and heterologous prime-boost strategies to mitigate the challenges of prior exposure and circulating vector-specific neutralizing antibodies and T-cells [23–25]. Rare serotype HAdV have been a focus of vaccination strategies targeting Ebola, with heterologous prime-boost vaccines initially explored using HAdV-26/HAdV-35 to minimize and bypass HAdV-5-related impairment through genetic segregation [26]. Most notably, the two-dose heterologous vaccine, Ad26.ZEBOV, has now received approval from the European Medicines Agency for emergency use in Ebola outbreaks, consisting of an HAdV-26 vectored vaccine and a modified vaccinia Ankara vector-based vaccine, both encoding Ebola virus glycoproteins [27–29]. More than 40 candidate adenoviral vector platforms have been assessed for safety and efficacy within Phase I-III clinical trials targeting a range of infectious diseases including zika virus, influenza and malaria [30–32]. To date, seven adenoviral vaccines have been licensed for use in humans [33], summarized in Table 1.

**Table 1:** Licensed adenoviral vector–based vaccines with regulatory approval for human use

Product	Vector serotype	Target	Encoded transgene	Delivery route	Year licensed	Ref.
ChAdOx1 nCoV-19 (AZD1222)	ChAdOx1	SARS-CoV-2	SARS–CoV–2 spike (S) protein	Intramuscular (IM)	2020 (UK)	[16,34]
Gam-COVID-Vac (Sputnik V)	HAdV-26 + HAdV-5	SARS-CoV-2	SARS–CoV–2 spike (S) protein	Intramuscular (IM)	2020 (Russia)	[35,36]
Ad5-nCoV (Convidecia)	HAdV-5	SARS-CoV-2	SARS–CoV–2 spike (S) protein	Intramuscular (IM)	2020 (China)	[9,37]
Ad26.COV2.S (Janssen/J&J)	HAdV-26	SARS-CoV-2	SARS–CoV–2 spike (S) protein	Intramuscular (IM)	2021 (USA)	[38,39]
Ad26.ZEBOV (Zabdeno)	HAdV-26	Ebola Zaire	EBOV glycoprotein (GP1/GP2)	Intramuscular (IM)	2020 (EU)	[40,41]
BBV154 (iNCOVACC)	ChAd36	SARS-CoV-2	SARS–CoV–2 spike (S) protein	Intranasal (IN)	2022 (India)	[42]
Ad5-nCoV-IH (Convidecia Air)	HAdV-5	SARS-CoV-2	SARS–CoV–2 spike (S) protein	Aerosol (AE)	2022 (China)	[43,44]

High transduction efficiency combined with large cassette capacity (>7.7 kbp) for transgene delivery initially made HAdV vectors attractive candidates for gene therapy [45]. However, immunogenicity against HAdV capsid proteins has limited research in this area, with undesirable host immunity from T-cells, neutralizing antibodies and innate activation detrimental to sustained transgene expression and efficient gene transfer [46]. Focus has now shifted toward the use of adeno-associated virus (AAV) vectors for therapeutic intervention within genetic disorders. In contrast to HAdVs, AAVs are nonpathogenic and associated with lower cell-mediated immunogenicity due to dampened innate immune sensing, despite high global seroprevelence [47–50]. Reduced host-AAV capsid immunogenicity is favourable for stable, long-term gene expression and tissue tropism of AVVs can be enhanced with the use of specific AAV serotypes, recently demonstrated following the approval of an AAV9-SMN1 gene replacement therapy (onasemnogene abeparvovec) for spinal muscular atrophy [51,52]. However, AAVs are restricted by a limited transgene packing capacity (4.7 kbp) and efficacy can be impeded by preexisting humoral immunity and antibodies directed against AVV capsid proteins [53,54]. Recently, work has been conducted to modify AAV capsid proteins to circumvent *de novo* immune responses. This was most notably performed by Bunning and colleagues who engineered a novel AAV2 serotype by introducing peptides (MyD88) into the AAV2 capsid to reduce innate and humoral immunogenicity whilst improving transduction efficiency [55].

Attempts to circumvent undesirable capsid immunogenicity also include the development of gutless adenovirus (GLAd) platforms, also referred to as helper dependent adenovirus vectors. GLAd have emerged as promising candidates for gene therapy and vaccine development, possessing minimal immunogenicity due to the deletion of viral coding sequences and removal of anti-vector immune responses that facilitate enhanced transduction efficiency and long-term transgene expression [56]. These third generation vectors offer several additional advantages, namely a larger cloning capacity (36–37 kbp) that allows for the delivery of transgene cargo capable of encoding more complex antigens including multigene cassettes and larger structural proteins [57,58]. However, as GLAd lack the adenoviral genome, production necessitates the incorporation of helper adenoviruses (first generation) which have proven difficult to fully remove from final viral preparations [59]. Contamination with classical E1/E3 deleted helper adenovirus leaves GLAd susceptible to the established barriers of preexisting immunity previously outlined and have hindered clinical use.

Potent innate and adaptive immunogenicity against adenoviral virion proteins can hinder applications against infectious diseases and inherited genetic disorders. However, these same immunological traits make HAdV attractive candidates for oncolytic virotherapies. Here, immunogenicity can drive inflammation and antitumour responses derived from the delivery of tumour associated neoantigens [60,61]. Oncolytic vectors can conditionally replicate within cancer cells and typically utilize a HAdV-5 backbone. Importantly, host exposure to viral capsid proteins can amplify the anticancer immune response following the release of pathogen-associated molecular patterns which serve to enhance dendritic cell function and cytotoxic CD8^+^ T-cell priming [62]. Several oncolytic HAdV-5 vectors have been assessed for efficacy against cancer in humans, such as CG0070 and DNX-2401 which selectively target bladder cancer and glioblastoma, respectively [63–66]. Attempts have also been made to introduce modified HAdV vectors with superior transduction efficiency, recently demonstrated by the development of pseudotyped vector incorporating HAdV-5 with a HAdV-35 fibre protein using a mouse cancer vaccine model [67^▪^].

### Preexisting immunity and immunogenicity of adenoviral delivery platforms

Adenovirus seroprevelence across human populations is of critical importance when selecting adenoviral platforms for transgene delivery. When screening for population level immunity against adenoviruses, it is also important to delineate prior exposure from functional neutralising antibody (NAb) responses, which can more accuracy reflect antibody-mediated vector interference and elimination through mechanisms such as opsonization, aggregation and complement activation [68]. Seminal seroepidemiology studies, performed by Barouch *et al.* assessed global titres of several HAdV serotypes to shed light on alternative vector candidates to HAdV-5. High NAb titres (>1 : 200) of emerging viral vector candidates, such as HAdV-26 (5–17%) and HAdV-35 (<5%), were found to be considerably lower than HAdV-5 (61–79%), although seroprevalence can vary by region [5,69]. Importantly, NAb responses are detectable across HAdV capsid proteins and studies have alluded to the dominance of anti-hexon titres compared to NAb responses directed against the penton base and fibre protein [70,71]. These findings, combined with studies demonstrating hexon hypervariable regions (HVR) are disproportionally (>90%) associated with neutralizing activity, provide a clear mechanistic target for capsid engineering in the form of HVR substitution and chimerization with rare seroprevelence HAdVs [72]. Furthermore, low human seroprevalence to nonhuman AdV, such as ChAd63, ChAd68 and ChAdY25 have been reported, which supported continued development as novel vaccine vector candidates that bypass humoral immunity [11,73–75].

**FIGURE 1:**
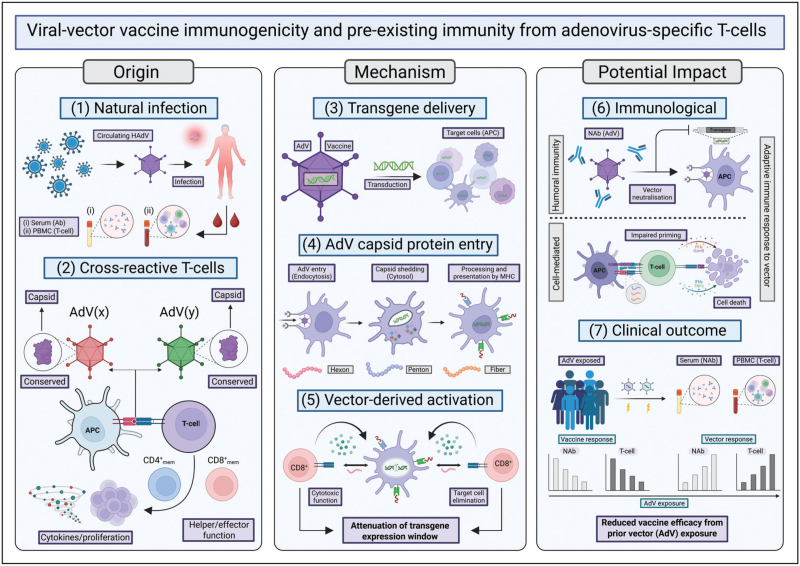
Impact of preexisting immunity to adenoviruses on the immunogenicity of viral-vector vaccines. (1) Immune responses to adenovirus (AdV) can originate from natural infection with circulating HAdV, resulting in detectable antibody responses (serum) and memory T-cell responses (PBMC) to AdV used in vector development. (2) Circulating adenovirus-specific T-cells can also be derived from T-cell cross-reactivity between AdV species due to the conserved nature of immunodominant epitopes contained within capsid proteins. (3) Viral genes needed for replication are replaced with a transgene encoding the antigen of interest. AdV vectors induce robust T-cell and B-cell immunogenicity following vector-mediated transduction into target cell population. AdV fibre knob proteins facilitate binding to Coxsackie–Adenovirus Receptors, enabling cellular transduction of antigen presenting cells (dendritic cells, monocytes and macrophages). (4) AdV cellular entry is mediated by endocytosis, and the transgene is delivered to the nucleus for transcription. Virion proteins (capsid) are released in the cell cytoplasm, leading to a spill over of viral peptides which are processed and presented on the surface of antigen presenting cells by MHC molecules. (5) T-cells recognise AdV peptides presented on MHC through immunological memory. Cytotoxic function of CD8^+^ T-cells can lead to vector-mediated target cell clearance and attenuation of the transgene expression window. (6) Immunological impact of preexisting adenoviral immunity. Neutralising antibodies (NAbs) against AdV can mediate vector interference and elimination through opsonisation, aggregation and complement activation. Cellular immune responses directed against AdV vector can impair T-cell priming to antigen of interest through disruption of transgene delivery and killing of antigen presenting cells. (7) Potential impact of preexisting AdV immunity on clinical responses to viral vector vaccines. Antibody and memory T-cell responses to AdV may correlate with viral exposure and enhance immunogenicity to AdV vectors while impeding vaccine/transgene-specific immune responses.

Despite limited cross-neutralization with HAdV-5 reported across HAdV species, T-cell responses are frequently detectable and broadly cross-reactive [11]. Unlike humoral immunogenicity derived from NAbs, preexisting T-cell immunity can occur independent of prior exposure due the conserved nature of adenoviral proteins contained within the hexon [76]. This is reinforced by studies detailing high frequencies of hexon-specific T-cells in the peripheral blood, with fibre-specific T-cell responses undetectable [77]. Analysis using German cohorts has confirmed this phenomenon, with >85% of donors possessing T-cells capable of being activated in the presence of HAdV-5 hexon or penton overlapping peptide pools [78]. T-cell cross-reactivity between adenoviral species and serotypes has now been well defined following the advent of rare serotype and non-human AdVs to which polyfunctional CD4^+^ and CD8^+^ T-cell responses have been detected in multiple cohorts [79,80]. This has most recently been evident in studies characterising T-cell responses to viral vector delivery platforms used in COVID-19 vaccination. Here, cross-reactive adenovirus-specific T-cell responses have been identified to viral particles of ChAdOx1 and structural proteins of ChAdY25 in PBMCs collected pre- and post-pandemic [81^▪^,82^▪^]. Cytotoxic CD8^+^ T-cell responses generated to HAdV-5 and ChAd capsid proteins have previously been shown to interfere with transgene delivery in animal models due to the direct clearance of vector transduced cells [83] Furthermore, data from clinical trials for the ChAdOx1-HBV vaccine conducted during the COVID-19 pandemic demonstrated that transgene-specific T-cell responses were diminished in individuals who had recently received ChAdOx1 nCoV-19 [84^▪▪^]. An overview of the immunological mechanisms by which preexisting immunity to AdV can influence the immunogenicity of viral-vector vaccines is displayed in Fig. 1.

Moving forwards, it will be important to define how anti-AdV immunity derived from cross-reactive T-cells affects vector suitability, thereby paving the way for capsid engineering to mitigate anti-vector immunogenicity whilst simultaneously preserving transduction efficiency and vaccine-specific immune responses. To this end, studies have identified several T-cell epitopes conserved across the adenoviral hexon that could be leveraged to develop HAdV vector candidates with reduced off-target immunogenicity against virion proteins. Olive *et al.* initially identified T-cell epitopes conserved across the hexon (H910–924) restricted to a common HLA-DP4 supertype and capable of serotypic cross-reactivity following the detection CD4^+^ T-cell responses in nearly 80% of individuals [85,86]. Furthermore, studies have also identified hexon epitopes associated with CD8+ T-cell activation located in the highly conserved C-terminus [87]. Hexon epitopes (H916–925) and hexon-derived peptides (TLLYVLFEV) are predominately restricted to the HLA-A*02 supertype and demonstrate T-cell cross-reactivity and killing capacity of vector-transduced host cells within PBMC assays [76,87,88]. However, research in this area has almost exclusively relied on *in silico* models of epitope binding prediction and synthetic peptide preparations. Such approaches lack immunopeptidomics validation and fail to capture the full repertoire of adenoviral peptides naturally presented by MHC. Therefore, it will now be crucial to delineate conserved adenoviral epitopes associated with antivector T-cell immunogenicity, incorporating MHC-associated peptide proteomics within developmental pipelines of new AdV vector candidates.

### Adverse reactions to adenoviral-based vaccines

Rare thrombotic events, known as vaccine-induced immune thrombotic thrombocytopenia (VITT), were the most severe adverse reaction documented following vaccination with replication-incompetent adenoviral vector-based COVID-19 vaccines. VITT is characterized by venous or arterial thrombosis and was first described after vaccination with ChAdOx1 nCoV-19 [89,90]. VITT can be further defined by high titres of platelet factor 4 (PF4) antibodies, with anti-PF4 antibodies clinically mimicking heparin-induced thrombocytopenia [91,92]. In rare cases, both ChAdOx1 nCoV-19 and Ad26.COV2. S were associated with the development of VITT, with ChAdOx1 nCoV-19 vaccination has been linked with a higher incidence (1/100 000) when compared to Ad26.COV2. S vaccination (1/300 000) [93]. Indeed, studies have indicated that adenoviral vector vaccination was responsible for approximately 98.5% of all VITT cases [94]. Mechanistically, there are a several factors that may influence VITT susceptibility and incidence after ChAdOx1 nCoV-19 vaccination. Multiple studies have demonstrated that the ChAdOx1 hexon protein can bind and form stable complexes with PF4 through electrostatic interactions [95,96]. This is possible due to the high electronegativity within the ChAdOx1 capsid and the cationic nature of PF4, with immune complexes inducing the production of anti-PF4 antibodies and subsequent platelet activation which can result in the release of neutrophil extracellular traps to facilitate clot formation [97,98]. Recently, several HAdV vectors that lack PF4 binding capacity have now been identified to mitigate thrombosis risk for future vaccine candidates [99]. T-cell involvement is well established in heparin-induced thrombocytopenia, for which pathomechanisms are closely aligned with VITT [100]. CD4^+^ T-cells have been found to play an important role in controlling PF4: heparin antibody production [101], with initial work eluding to the requirement of helper T-cells for antibody class switching through the recognition of peptides derived from PF4 [102]. Therefore, it is reasonable to hypothesise that circulating T-cells may actively contribute to VITT development, with further investigation needed to examine the role of anti-vector T-cell responses within rare thrombotic events in patients with clinically defined VITT.

Adenoviral COVID-19 vaccines have also been associated with a range of cutaneous-based adverse reactions, encompassing mild injection site manifestations and more severe, T-cell mediated adverse events [103]. Delayed-type, T-cell mediated hypersensitivity (Type IV) represents the second most prevalent adverse event after vaccination, with reactions manifesting between 3 and 21 days post intramuscular injection [103,104]. Type IV hypersensitivity is antibody independent and can be derived from aberrant T-cell stimulation and resultant secretion of effector molecules associated with cytotoxicity, inflammation and tissue damage which are contributing factors for severe cutaneous adverse reactions (SCAR). Triggers of an immune aetiology can include signalling from type 1 interferons derived from intrinsic vaccine immunogenicity and the activation resident preexisting memory T-cells combined with immunoregulatory imbalance [105]. Pustular drug reactions, such as acute generalized exanthematous pustulosis (AGEP), have been reported following exposure to viral-vector vaccines [106]. Work published by Lospinoso *et al.* identified a case of drug reaction with eosinophilia and systemic symptoms (DRESS) with AGEP overlap after Ad26.COV2.S administration, with SCAR diagnosis supported by marked eosinophilia and confirmed by histopathological analysis of a skin biopsy [107]. Rare instances of Stevens-Johnson syndrome (SJS)/toxic epidermal necrolysis (TEN), a life-threatening mucocutaneous reaction characterized by the degree epidermal detachment from the dermis [108], have been reported following ChAdOx1 nCoV-19 vaccination. Here, case studies confirmed SJS presentation through the identification of subepidermal blistering and epidermal necrosis 3  weeks after the first dose of ChAdOx1 nCoV-19, consistent with delayed-type hypersensitivity [109]. Recent case series have now suggested a potential association between SJS/TEN, SARS-CoV-2 infection and a range of viral vector-based COVID-19 vaccines [110^▪^]. Although exact pathomechanisms are unclear, it is possible that adenoviral proteins may directly bind to immunological receptors in a similar fashion to low molecular weight therapeutic compounds. In this way, cytotoxic T-cell responses may be triggered through granule-mediated pathways, driving keratinocyte cell death and SJS/TEN pathogenesis [111]. Furthermore, the concept of SCAR presentation arising from viral reactivation stemming from SARS-CoV-2 infectivity is also important to consider within the context of SJS and DRESS. It is widely accepted that viral proteins can bind MHC complexes on the surface of antigen presenting cells to facilitate cytotoxic function. Indeed, delayed antiviral CD8+ T-cell responses have been observed in SCAR patients infected with human herpes viruses, Epstein-Barr virus and cytomegalovirus [112–114]. For this reason, it can be difficult ascertain causative agents within severe cutaneous reactions due to the eitopathogenic overlap that exists between exposure to viral-vector vaccines and viral infection. However, it must be stated that SCAR incidence following vaccination is extremely rare, with any risk greatly outweighed by diseases protection offered by viral vector-based vaccines [115,116^▪▪^]. In the case of ChAdOx1 nCoV-19, severe T-cell mediated adverse events were not encountered during phase 2 and 3 clinical trials [117]. A summary of adenoviral vaccine-mediated adverse events associated with an immune aetiology can be viewed in Table 2.

**Table 2:** Immune-mediated adverse events reported after vaccination with adenoviral-vector vaccines

Adverse event	Clinical features	Onset	Adenoviral vaccine(s)	Reference
Localised reactogenicity				
Injection site reactions	Pain, swelling, erythema or induration at site of IM injection (1–10% of vaccine recipients).	<48 h post administration.	ChAdOx1 nCoV-19, Ad26.COV2.S, Ad5-nCoV	[38,118–120]
Delayed local reactions	Large local cutaneous reaction. Pain, pruritus, warm sensation and erythema multiforme.	Typical onset 4–16 days post IM injection of vaccine.	ChAdOx1 nCoV-19	[121,122]
Systemic reactogenicity				
Generalised systemic reactogenicity (solicited)	Fatigue, fever, nausea, malaise, myalgia. Self-limiting (>10% of vaccine recipients).	<48 h post IM injection of vaccine.	ChAdOx1 nCoV-19, Ad26.COV2.S, Ad5-nCoV	[38,118–120]
Neurological	Headache, dizziness, nausea (self-limiting/transient).	<48 h post IM injection of vaccine.	ChAdOx1 nCoV-19, Ad26.COV2.S, Ad5-nCoV	[9,38,117]
Immediate hypersensitivity reactions (type I)				
Urticaria and angioedema	Hives and deep swelling of the dermis. IgE-mediated, mast cell degranulation leads to release of proinflammatory mediators (histamine).	Minutes after vaccination.	Excipients: Polysorbate 80 (ChAdOx1 nCoV-19, Ad26.COV2.S).Potential cross-reactivity with polyethylene glycol reported.	[123,124]
Anaphylaxis	Severe, IgE-mediated adverse reaction. Rapid release of inflammatory mediators from mast cells and basophils.	>85% of anaphylaxis cases triggered by COVID-19 vaccines occur within 30 min.	ChAdOx1 nCoV-19, Ad26.COV2.S, Ad5-nCoV (also reports with mRNA vaccine platforms).	[125,126]
Severe cutaneous adverse reactions (SCAR)				
Stevens-Johnson syndrome/toxic epidermal necrolysis (SJS/TEN)	Life-threatening CD8^+^ T-cell mediated reaction with mucosal involvement, characterised by degree of epidermal detachment (SJS, <10%; TEN, >30%).	10 days–3 weeks after first vaccine dose. Confirmed by skin biopsy (subepidermal blistering/necrosis).	ChAdOx1 nCoV-19 (also reports with mRNA vaccine platforms).	[109,127,128]
Acute generalized exanthematous pustulosis (AGEP) and drug reaction with eosinophilia and systemic symptoms (DRESS)	T-cell mediated (CD4^+^/CD8^+^) pustular drug reaction. Generalized distribution of erythematous plaques and nonfollicular pustules	3 days after first vaccine dose. Eosinophilia, confirmed by skin biopsy	Ad26.COV2.S	[107]
Haematological reactions (immune-mediated)				
Vaccine-induced immune thrombotic thrombocytopenia (VITT)	Venous or arterial thrombosis. Severe, antibody mediated. Characterized by high titres of PF4 antibodies.	5–20 days following vaccination.	ChAdOx1 nCoV-19 (1/100 000) Ad26.COV2.S (1/300 000)	[90–93,98]

## CONCLUSION

Rapid vaccine development is a critical part of a pandemic response, and there are many endemic diseases for which there is no effective, safe and easy to deliver vaccine. Preexisting immunity to adenoviral vectors has long been a challenge within vaccine development. Initially, concerns surrounded the future suitability of HAdV-5, stemming from high seroprevalence rates that can attenuate vector efficacy. Attempts to circumvent preexisting immunity following the advent low seroprevalence adenoviral vectors may encounter similar challenges, with multiple studies now defining the broadly cross-reactive nature of T-cell responses across species and serotypes, absent serological exposure. Widespread use of adenoviral vector-based vaccines during the COVID-19 pandemic has further expanded population-level cellular immunity to these platforms. While essential for pandemic control, the long-term implications for the use for the use of adenoviral systems for future vector applications remain incompletely understood. There is now evidence that future applications of adenoviral vectors will require rational re-engineering to evade vaccine and vector-induced cellular immunity.

## Acknowledgements

*None.*

### Financial support and sponsorship

*This work was supported by a Wellcome Early Career Award (321990/Z/24/Z).*

### Conflicts of interest

*There are no conflicts of interest.*

## REFERENCES AND RECOMMENDED READING

Papers of particular interest, published within the annual period of review, have been highlighted as:

▪ of special interest
▪▪ of outstanding interest
